# Association of *ADIPOR2 *gene variants with cardiovascular disease and type 2 diabetes risk in individuals with impaired glucose tolerance: the Finnish Diabetes Prevention Study

**DOI:** 10.1186/1475-2840-10-83

**Published:** 2011-09-24

**Authors:** Niina Siitonen, Leena Pulkkinen, Jaana Lindström, Marjukka Kolehmainen, Ursula Schwab, Johan G Eriksson, Pirjo Ilanne-Parikka, Sirkka Keinänen-Kiukaanniemi, Jaakko Tuomilehto, Matti Uusitupa

**Affiliations:** 1 Department of Clinical Nutrition and Food and Health Research Centre, Institute of Public Health and Clinical Nutrition, University of Eastern Finland, Kuopio, Finland; 2 Department of Health Promotion and Chronic Disease Prevention, National Institute for Health and Welfare, Helsinki, Finland; 3 Department of Public Health, University of Helsinki, Helsinki, Finland; 4 Institute of Medicine, Clinical Medicine, Kuopio University Hospital, Kuopio, Finland; 5 Folkhalsan Research Centre, Helsinki, Finland; 6 Department of General Practice and Primary Health Care, University of Helsinki, Helsinki, Finland; 7 Vasa Central Hospital, Vasa, Finland; 8 Unit of General Practice, Helsinki University Central Hospital, Helsinki, Finland; 9 Diabetes Centre, Finnish Diabetes Association, Tampere, Finland; 10 Science Centre, Pirkanmaa Hospital District, Tampere University Hospital, Tampere, Finland; 11 Institute of Health Sciences, University of Oulu, Oulu, Finland; 12 Unit of General Practice, Oulu University Hospital, Oulu, Finland; 13 Health Centre of Oulu, Oulu Finland; 14 South Ostrobothnia Central Hospital, Seinäjoki, Finland; 15 Department of Preventive and Clinical Medicine, Danube-Universität Krems, Krems, Austria; 16 Research Unit, Kuopio University Hospital, Kuopio, Finland

**Keywords:** Adiponectin, adiponectin receptor 2, gene, single nucleotide polymorphism, cardiovascular disease, type 2 diabetes

## Abstract

**Background:**

Adiponectin is an adipokine with insulin-sensitising and anti-atherogenic effects. Two receptors for adiponectin, ADIPOR1 and ADIPOR2, have been characterized that mediate effects of adiponectin in various tissues. We examined whether genetic variation in *ADIPOR2 *predicts the development of cardiovascular disease (CVD) and/or Type 2 Diabetes (T2DM) in individuals with impaired glucose tolerance (IGT) participating the Finnish Diabetes Prevention Study (DPS).

**Methods:**

CVD morbidity and mortality data were collected during a median follow-up of 10.2 years (range 1-13 years) and conversion from IGT to T2DM was assessed during a median follow-up of 7 years (range 1-11 years). Altogether eight SNPs in the *ADIPOR2 *locus were genotyped in 484 participants of the DPS. Moreover, the same SNPs were genotyped and the mRNA expression levels of *ADIPOR2 *were determined in peripheral blood mononuclear cells and subcutaneous adipose tissue samples derived from 56 individuals participating in the Genobin study.

**Results:**

In the DPS population, four SNPs (rs10848554, rs11061937, rs1058322, rs16928751) were associated with CVD risk, and two remained significant (p = 0.014 for rs11061937 and p = 0.020 for rs1058322) when all four were included in the same multi-SNP model. Furthermore, the individuals homozygous for the rare minor alleles of rs11061946 and rs11061973 had increased risk of converting from IGT to T2DM. Allele-specific differences in the mRNA expression levels for the rs1058322 variant were seen in peripheral blood mononuclear cells derived from participants of the Genobin study.

**Conclusions:**

Our results suggest that SNPs in the *ADIPOR2 *may modify the risk of CVD in individuals with IGT, possibly through alterations in the mRNA expression levels. In addition an independent genetic signal in *ADIPOR2 *locus may have an impact on the risk of developing T2DM in individuals with IGT.

**Trial registration number:**

ClinicalTrials.gov NCT00518167

## Background

Adipose tissue (AT) secretes a number of bioactive molecules, called adipokines, which participate in regulation of various metabolic processes [1]. Excess adiposity, particularly central type of adiposity, is associated with chronic low grade inflammation and dysregulated production of adipokines with adverse metabolic consequences, such as insulin resistance, hypertension, dyslipidemia, and increased risk of cardiovascular disease (CVD) [1].

Adiponectin is an adipokine with insulin-sensitising, anti-inflammatory and anti-atherogenic properties [2-7]. Circulating levels of adiponectin are decreased in obesity [8], dyslipidemia [9], cardiovascular disease (CVD) [10], insulin resistance and T2DM [8]. The insulin-sensitising effects of adiponectin involve stimulation of fatty acid oxidation and glucose uptake in skeletal muscle and suppression of gluconeogenesis in liver [5]. The anti-atherogenic effects of adiponectin include suppression of adhesion molecule expression on vascular endothelial cells [6], and inhibition of vascular smooth muscle cell proliferation and migration [7]. Adiponectin also stimulates the production of NO in endothelial cells [11] and reduces atherosclerosis by suppressing endothelial inflammatory reaction and macrophage to foam cell transformation [2].

Two adiponectin receptors, ADIPOR1 and ADIPOR2, have been characterized that mediate the functions of adiponectin through activation of 5' AMP-activated protein kinase (AMPK) and peroxisome proliferator-activated receptor alpha (PPARα) [12]. In mice, *AdipoR2 *is mostly expressed in liver, while *AdipoR1 *is ubiquitously expressed [12]. Mice deficient for either or both receptors were glucose intolerant, and adenovirus mediated expression of *AdipoR1 *and *AdipoR2 *in liver improved obesity-related insulin resistance and T2DM in *db/db *mice through AMPK and PPARα pathways, respectively [13]. In contrast, Bjursel et al. reported that *AdipoR2*-KO mice were lean and resistant to high fat diet induced weight gain, had improved glucose tolerance and decreased plasma total and HDL cholesterol levels [14]. In humans, *ADIPOR2 *is highly expressed in skeletal muscle and correlates positively with insulin sensitivity [15] and fasting plasma triglyceride concentrations in healthy glucose tolerant subjects [16]. On the contrary, the expression level of *ADIPOR2*, but not *ADIPOR1*, was decreased in the intra-abdominal AT of obese individuals, and correlated negatively with triglyceride and apolipoprotein B levels [17]

*ADIPOR2 *gene is located on chromosomal locus 12p13.33 and consists of nine exons. Associations between *ADIPOR2 *gene variants with insulin resistance and T2DM related phenotypes [18-21], triglyceride levels [22-24], liver fat content [24,25], and CAD [26] have been reported in several human populations. Other studies have, however, failed to detect associations between *ADIPOR2 *variants and T2DM [27-29] or other metabolic parameters [30,31]. The differences may be attributed to different characteristics of study participants or differences in study designs.

Most previous studies investigating the role of *ADIPOR2 *variants in T2DM and related phenotypes have been cross-sectional, and therefore have not been able to evaluate the effect of *ADIPOR2 *variants in the development of T2DM. The aim of this study was to investigate the role of *ADIPOR2 *locus variation in individuals with impaired glucose tolerance (IGT) participating in a controlled lifestyle intervention study (DPS study) with longitudinal data on metabolic and anthropometric parameters, and CVD incidence. In addition, we analysed whether *ADIPOR2 *variants were associated with the mRNA expression levels in PBMCs and subcutaneous AT samples derived from individuals with metabolic syndrome.

## Methods

### DPS population and study design

DPS is a randomised, controlled multicentre study with five participating study clinics in Finland. The main aim of DPS was to assess the efficacy of lifestyle modification on preventing or delaying the onset of T2DM in individuals with IGT. The diagnosis of IGT and T2DM were based on WHO 1985 criteria [32]. IGT (fasting plasma glucose < 7.8 mmol/l and a 2-h plasma glucose 7.8-11.0 mmol/l) was based on the mean value of two oral glucose tolerance tests (OGTT) and diagnosis of T2DM was confirmed by a second OGTT [33]. The study design has been described earlier in detail [33,34]. Altogether 522 overweight (BMI≥25 kg/m^2^) volunteers aged 40-64 years were randomly allocated into an intensive diet and exercise intervention group or a control group. The individuals in the intervention group received individualized diet and exercise counseling, whereas individuals in the control group were given general information on healthy diet and exercise [35]. The median length of the intervention period was four years (range 1-6 year).

### Clinical and biochemical analyses in DPS

At baseline and annually, a medical history was recorded and physical examination with anthropometric measurements performed on each study participant. In addition, a 2-hour OGTT was performed annually with glucose a load of 75 g. Plasma glucose was measured locally by standard methods as previously described [35]. Serum insulin was measured by RIA (Phadaseph Insulin RIA 100, Pharmacia Diagnostica, Uppsala, Sweden). The intra-assay coefficient of variation was 5.3% and the interassay coefficient of variation was 7.6%. Concentrations of serum total and HDL cholesterol, and serum triglycerides were determined annually from fasting samples by using an enzymatic assay method in central laboratory in Helsinki. LDL cholesterol concentrations were calculated by using the Friedewald formula.

Mortality and cardiovascular morbidity data were collected after a median 10.2 years (range 1-13 years) follow-up of from the national Hospital Discharge Register and Causes of Death Register using the unique personal identification number [36]. The end-points during follow-up were total mortality, and incident cardiovascular events (fatal and non-fatal), including acute coronary events, coronary heart disease, stroke and hypertensive disease.

Frozen serum samples for adiponectin measurements were only available from a subset of participants from three study clinics at baseline (n = 243). Fasting serum adiponectin levels were measured using an enzyme-linked immunosorbent assay (ELISA) (B-Bridge International, Inc., San Jose, CA, USA), on whole plasma stored at -80°C. The intra-assay and inter-assay coefficients of variation were 5.5-7.9% and 6.5%, respectively.

The study protocol was approved by the Ethics Committee of the National Public Health Institute in Helsinki, Finland. Written informed consent was received from all participants [33,34]. We certify that all applicable institutional and governmental regulations concerning the ethical use of human volunteers were followed during this study.

### Genobin study population and study design

Seventy-five overweight or obese (BMI 28-40 kg/m^2^) men and women (aged 40-70 years) were recruited to the study. The subjects had impaired fasting glycemia (IFG: fasting plasma glucose concentration 5.6-7.0 mmol/l), or IGT (2-h plasma glucose concentration 7.8-11.0 mmol/l and fasting plasma glucose <7.1 mmol/l), and fulfilled at least two criteria of the metabolic syndrome according to the Adult Treatment Panel III Criteria [37] as modified by the American heart Association [38]: waist circumference >102 cm for men and >88 cm for women; fasting serum triacylglycerol ≥1.7 mmol/l; fasting serum HDL cholesterol <1.0 mmol/l for men and <1.3 for women; blood pressure ≥130/80 mmHg. The Ethics Committee of the District Hospital Region of Northern Savo approved the study plan. All participants volunteered for the study and gave their written informed consent.

### Analysis of gene expression from PBMCs and AT samples

Peripheral blood mononuclear cells (PBMCs) were isolated from anticoagulated peripheral blood, collected at baseline, by using Lymphoprep reagent (Axis-Shield, Oslo, Norway). A needle biopsy was taken after an overnight fast from subcutaneous abdominal AT under local anesthesia (Lidocaine 10 mg/ml without epinephrine) at baseline. The AT samples for the RNA extraction were treated with RNA later according to instructions provided by manufacturer (Ambion, Austi, TX, USA) and stored at -80°C.

Altogether 56 baseline PBMC and AT samples were available. Total RNA was extracted using an RNeasy Mini Kit (Qiagen, Valencia, CA, USA) according to the instructions of manufacturers. RNA was reverse transcribed into cDNA by using High-Capacity cDNA Archive Kit (Applied Biosystems, Foster City, CA, USA). RNA concentration and A_260_/A_280 _ratio were measured using NanoDrop spectrophotometer (Nanodrop Technologies, Wilmington, DE) with acceptable ratio being 1.9-2.1 Integrity of RNA sample was assessed using agarose gel electrophoresis.

Real-time PCR was performed with ready-made assays based on TaqMan chemistry and analysed with ABI Prism 7500 SDS software (Applied Biosystems). All samples were analysed as triplicates and each reaction consisted of 6 ng cDNA, 1X Assay Mix, and 1X Taqman Universal PCR Master Mix (Applied Biosystems). The relative quantity of cDNA in each sample was assessed by using the methods described in ABI Prism User Bulletin no. 2. Briefly, on each plate, a standard curve with five known cDNA concentrations (0.5, 1.5, 6, 18, and 36 ng/μl) and a calibrator (6 ng/μl) were included. The cDNA quantity of each sample was determined by comparing it to the standard curve and the relative quantity was calculated by dividing it with the quantity of calibrator. Finally, the relative expression quantity on each plate was normalised to the expression levels of an endogenous control gene, which was *glyceraldehydes-3-phosphate dehydrogenase *for the PBMCs and *cyclophilin A1 *for the AT samples.

### Selection of SNPs and genotyping

DNA samples were available from 484 DPS participants (160 men and 324 women) and 56 Genobin participants (29 men and 27 women). Tagging SNPs were selected based on genotype data of the Hapmap CEU (Utah residents with ancestry from Northern and Western Europe) population [39] by using the Tagger algorithm [40]. The eight SNPs selected for genotyping in DPS captured 63% (60 of 94) of common variants in the *ADIP*OR2 locus with r^2 ^≥ 0.8. All SNPs were genotyped with TaqMan Allelic Discrimination assays according to manufacturer's instructions by using the ABI PRISM 7000 sequence detector (Applied Biosystems, Foster City, CA). For a subset of randomly selected samples (6.3%) genotyping was repeated in order to calculate success rate.

### Statistics

Statistical tests were performed using the SPSS statistical software for Windows (version 14.0, SPSS Inc., Chigago, IL, USA). The pairwise linkage disequilibrium (LD) between SNPs in DPS was evaluated with Haploview software (version 4.2; Broad institute, Cambridge, MA) [41].

The Hardy-Weinberg equilibrium (HWE) and genotype distributions among study groups were analysed using Chi-square test. The normality of distributions of the continuous variables was checked with the Kolmogorov-Smirnov test with Lilliefors correction and variable transformations were applied when appropriate. Genetic analyses were carried out using additive (all three genotype groups were compared) or dominant (common allele homozygotes were compared with the minor allele carriers) inheritance models. Using appropriate adjustments, the genotype differences in continuous variables were analysed with general linear model (GLM) univariate analysis of variance (ANOVA). Normality was assessed by plotting the residuals.

The association of *ADIPOR2 *SNPs with cardiovascular risk was analysed with Cox regression model adjusting for age, sex, baseline waist circumference, study group, CVD history at baseline, systolic blood pressure smoking status, and total-to-HDL cholesterol ratio at baseline in 467 DPS participants for whom CVD data and the relevant covariates were available. Likewise, in 482 participants for whom all relevant variables were available the association of the *ADIPOR2 *SNPs with conversion to T2DM was analysed by using Cox regression adjusting for age, sex, waist circumference, study group, and either fasting or 2-hour plasma glucose.

Multiple hypothesis testing is a general problem in genetic association studies and increases the likelihood of false positive findings. In order to control for multiple testing, we used the false discovery rate (FDR) method with the Q value 1.0 software [42] for single SNP analyses and provide q-values in combination with p-values to help in the interpretation of the results. The q-values were calculated separately for each trait, but all SNPs and both inheritance models were included in the same calculations. Level of statistical significance was set to p < 0.05, and the q-values of q < 0.150 were considered as additional proof for true associations.

## Results

### Baseline characteristics and genotype frequencies

Table 1 shows the clinical and metabolic baseline characteristics for both study populations. Eight *ADIPOR2 *SNPs were genotyped from the DNA samples of 484 DPS and 56 Genobin participants with an error rate of 0% and a call rate of 100% in replicated samples for all markers. Genotype counts and minor allele frequencies are reported in table 2 and the pairwise LD measures in the DPS population are presented in table 3. All SNPs, except rs11061946 in the DPS, were in Hardy-Weinberg equilibrium (HWE, p > 0.05). However, we included this SNP in further analyses, since the q-value was high (q > 0.150) and the deviation from HWE was likely a chance finding resulting from small minor allele frequency (MAF). Two SNPs (rs11061946 and rs11061973) demonstrated differential genotype frequencies in the DPS study groups according to p-value (p < 0.05), but not according to q-value (q > 0.150). We included these SNPs in all analyses, but performed all analyses concerning the follow-up data separately in both groups.

**Table 1:** Baseline characteristics of the DPS and Genobin study participants

	DPS	Genobin
Males (n)/Females (n)	160/324	29/27
Age (y)	55.2 ± 7.0 (484)	59.4 ± 6.8 (56)
Weight (kg)	86.3 ± 14.2 (484)	92.8 ± 14.0 (56)
BMI (kg/m^2^)	31.2 ± 4.5 (484)	32.7 ± 2.9 (56)
Waist circumference (cm)	101.2 ± 11.0 (482)	108.9 ± 8.9 (56)
Fasting plasma glucose (mmol/L)	6.13 ± 0.75 (484)	6.44 ± 0.49 (56)
2-h plasma glucose (mmol/L)	8.88 ± 1.49 (484)	7.42 ± 2.15 (56)
Fasting serum insulin (mU/L)	14.73 ± 7.46 (439)	11.96 ± 7.29 (56)
2-h serum insulin (mU/L)	95.57 ± 65.83 (436)	83.38 ± 72.92 (56)
Serum total cholesterol (mmol/L)	5.61 ± 0.93 (483)	5.16 ± 0.98 (56)
Serum HDL cholesterol (mmol/L)	1.21 ± 0.29 (483)	1.23 ± 0.22 (56)
Serum LDL cholesterol (mmol/L)	3.62 ± 0.84 (481)	3.37 ± 0.91 (56)
Serum triglycerides (mmol/l)	1.73 ± 0.78 (483)	1.66 ± 0.84 (56)
Diastolic blood pressure (mmHg)	86 ± 10 (479)	89 ± 10 (56)
Systolic blod pressure (mmHg)	138 ± 17 (479)	136 ± 14 (56)

Data are mean ±SD

**Table 2:** Genotype counts and allele frequencies of *ADIPOR2 *SNPs in the DPS and Genobin study population

SNP and its location within gene						HWE	
	Population	Genotype counts			MAF	p/q^a^	p/q^b^
rs10848554		GG	GC	CC	C		
5' promoter region	DPS	373	104	7	0.122	0.934/0.389	0.401/0.204
	Genobin	44	12	0	0.107	0.369/0.377	

rs11061937		TT	TC	CC	C		
Intron 1	DPS	213	214	57	0.339	0.772/0.378	0.208/0.170
	Genobin	16	27	13	0.473	0.805/0.378	

rs11061946		CC	CT	TT	T		
Intron 1	DPS	430	49	5	0.061	0.011/0.377	0.042/0.170
	Genobin	48	8	0	0.071	0.565/0.377	

rs1058322		CC	CT	TT	T		
Intron 1	DPS	230	206	48	0.320	0.850/0.378	0.958/0.299
	Genobin	22	29	5	0.348	0.292/0.377	

rs11061973		GG	GA	AA	A		
Intron 2	DPS	403	76	5	0.089	0.508/0.377	0.027/0.170
	Genobin	44	12	0	0.107	0.369/0.377	

rs4766415		AA	AT	TT	T		
Intron 2	DPS	128	254	102	0.473	0.247/0.377	0.243/0.170
	Genobin	11	26	19	0.429	0.697/0.378	

rs16928751		GG	GA	AA	A		
Exon 7	DPS	379	101	4	0.113	0.331/0.377	0.493/0.206
	Genobin	47	9	0	0.080	0.513/0.377	

rs1044471		CC	CT	TT	T		
3' UTR	DPS	136	245	103	0.466	0.706/0.378	0.578/0.207
	Genobin	22	28	6	0.357	0.506/0.377	

^a^p/q value for Hardy-Weinberg equilibrium; ^b^p/q value for differences in genotype frequencies between the study groups of DPS; Correction for multiple hypothesis testing was performed with FDR, denoted as q-value.

**Table 3:** Pairwise linkage disequilibrium measures of the *ADIPOR2 *SNPs in the DPS population

	SNP1	SNP2	SNP3	SNP4	SNP5	SNP6	SNP7	SNP8
SNP1		1.0	0.483	0.971	1.0	1.0	1.0	1.0
SNP2	0.071		1.0	0.101	0.977	0.955	1.0	0.945
SNP3	0.0020	0.125		0.291	1.0	1.0	0.314	1.0
SNP4	0.291	0.0090	0.012		0.094	0.637	0.984	0.569
SNP5	0.013	0.178	0.674	0.0020		1.0	0.912	1.0
SNP6	0.155	0.525	0.072	0.204	0.107		1.0	0.922
SNP7	0.918	0.065	0.0010	0.275	0.01	0.143		1.0
SNP8	0.121	0.401	0.056	0.127	0.083	0.663	0.111	

SNP1, rs10848554; SNP2 rs11061937; SNP3, rs11061946; SNP4, rs1058322; SNP5, rs11061973; SNP6, rs4766415; SNP7, rs16928751; SNP8, rs1044471; D' (above empty cells) and r^2 ^(below empty cells)

### Risk of cardiovascular event

**Figure 1:**
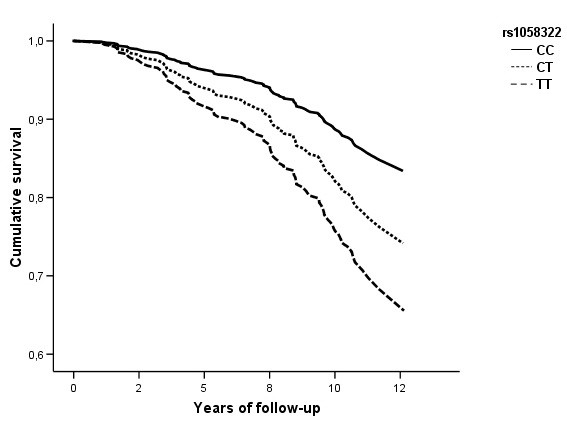
**Survival curve for CVD incidence according to *ADIPOR2 *rs1058322 in DPS participants**. Additive inheritance model, adjusted for age, sex, baseline waist circumference, study group, CVD at baseline, systolic blood pressure, smoking status, and baseline total-to-HDL cholesterol ratio.

After a median follow-up period of 10.2 years, there were 100 CVD events among the DPS participants included in these analyses (50/241 in the intervention and 50/232 in the control group) [36]. Of the eight *ADIPOR2 *SNPs included in the analyses, differences in the CVD risk were seen according to four SNPs (table 4 and figure 1 for rs1058322). The rs10848554 C allele was associated with an increased risk of CVD when compared with the GG genotype (p = 0.048/q = 0.095). Moreover, individuals carrying the rs11061937 C allele had significantly lower risk of CVD events compared with those with the TT genotype (p = 0.013/q = 0.095). When all three genotypes were compared, the risk was lower for individuals with the TC genotype compared with those with the TT genotype (p = 0.012/q = 0.095). Finally, the rs1058322 T allele and the rs16928751 A allele were dose-dependently associated with higher risk of CVD (p = 0.014/q = 0.095 and p = 0.015/q = 0.095, respectively for the dominant inheritance model). Although significant study group-SNP interaction was not observed for any of the SNPs, we performed all analyses separately in both study groups and found similar results in both (data not shown). Moreover, we did not find significant interaction between *ADIPOR2 *variants and sex or baseline BMI on CVD risk.

**Table 4:** Hazard ratios for the significant association between *ADIPOR2 *SNPs and the development of CVD during follow-up of median 10.2 years

		HR (95% CI), p/q^a^	HR, 95% CI, p/q^b^
rs10848554	GG (358)	1	1
	GC (102)	1.510 (0.957-2.382), 0.076/0.110	1.565 (1.004-2.440), 0.048/0.095
	CC (7)	2.919 (0.697-12.23), 0.143/0.150	

rs11061937	TT (207)	1	1
	TC (206)	0.568 (0.364-0.885), 0.012/0.095	0.595 (0.395-0.896), 0.013/0.095
	CC (54)	0.701 (0.353-1.390), 0.309/0.238	

rs1058322	CC (218)	1	1
	CT (201)	1.601 (1.021-2.509), 0.040/0.095	1.711 (1.114-2.627), 0.014/0.095
	TT (48)	2.300 (1.194-4.433), 0.013/0.095	

rs16928751	GG (364)	1	1
	GA (99)	1.677 (1.051-2.674), 0.030/0.095	1.756 (1.114-2.766), 0.015/0.095
	AA (4)	4.425 (1.042-18.79), 0.044/0.095	

All analyses were adjusted for age, sex, baseline waist circumference, baseline 2-h plasma glucose and study group; Correction for multiple hypothesis testing in single SNP analyses was performed by using FDR, denoted as q-value; ^a^Additive inheritance model for single SNP analyses; ^b^Dominant inheritance model for single SNP analyses.

In a multiple SNP-model, including all four SNPs that individually were associated with CVD risk, two variants (rs11061937 and rs1058322) remained significant predictors of CVD risk (p = 0.016 and p = 0.023, respectively).

### CVD risk and baseline serum adiponectin levels

In a subgroup of DPS participants whose serum adiponectin levels were measured at baseline, we analysed whether circulating adiponectin levels predict future risk of CVD. A trend for lower CVD risk was seen in those with higher than the median (6.250 for men and 8.815 for women) serum adiponectin levels, compared with those with adiponectin below the median (HRR 0.564, 95% CI 0.312-1.019, p = 0.058).

### The risk of T2DM

During the median follow-up of 7 years (range 1-11 years), the number of new T2DM cases among the DPS participants included in these analyses was 175 (71/245 in the intervention group and 104/239 in the control group) [43]. Subjects homozygous for rs11061946 and rs11061973 minor alleles (n = 5), had significantly higher risk of T2DM compared with subjects homozygous for the major alleles of these SNPs when adjusted for age, sex, study group, baseline waist circumference, and either fasting or 2-h glucose levels. The results are presented in table 5 for model adjusted with 2-h glucose levels, but the results were essentially similar when the model was adjusted for fasting plasma glucose instead. When the dominant inheritance model was used, no genotype differences in risk of T2DM were observed. When study groups were analyzed separately, results were similar in both study groups (data not shown).

**Table 5:** Hazard ratios for the significant association between *ADIPOR2 *SNPs and the development of T2DM during follow-up of median 7 years

		HR (95% CI), p/q^a^	HR (95% CI), p/q^b^
rs11061946	CC (428)	1	1
	CT (49)	0.706 (0.412- 1.211), 0.206/0.517	0.872 (0.536- 1.417), 0.579/0.596
	TT (5)	5.538 (2.014- 15.226), 0.001/0.369	

rs11061973	GG (401)	1	1
	GA (76)	0.951 (0.636- 1.423), 0.808/0.657	1.061 (0.724- 1.557), 0.761/0.657
	AA (5)	5.683 (2.066- 15.636), 0.001/0.369	

All analyses were adjusted for CVD at baseline, age, sex, baseline waist circumference, baseline total to HDL cholesterol ratio, smoking status, systolic blood pressure and study group; Correction for multiple hypothesis testing in single SNP analyses was performed by using FDR, denoted as q-value; ^a^Additive inheritance model for single SNP analyses; ^b^Dominant inheritance model for single SNP analyses.

### ADIPOR2 mRNA expression in the Genobin study population

**Figure 2:**
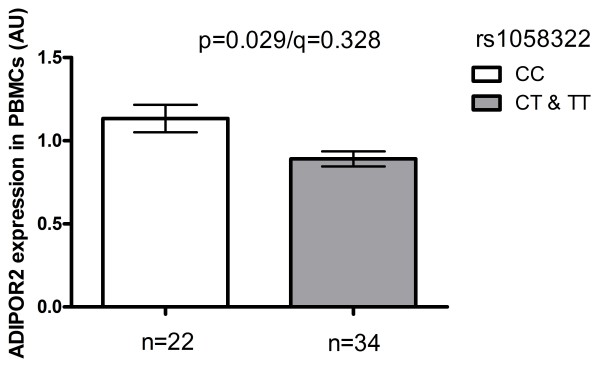
**The mRNA expression of *ADIPOR2 *in PBMCs of Genobin study participants according to rs1058322**. Values are means ± SEM. Dominant inheritance model, adjusted for age, sex and baseline BMI.

The *ADIPOR2 *mRNA expression in the PBMCs differed according to rs1058322 genotype (figure 2) at baseline (p = 0.029/q = 0.328 for the dominant inheritance model adjusted for age, sex and baseline BMI). Individuals carrying the T allele had lower expression levels compared with those with the CC genotype. Other SNPs in *ADIPOR2 *did not associate with mRNA expression levels in PBMCs and differences in expression levels according to ADIPOR2 SNPs were not observed in AT.

## Discussion

The results of the present study suggest that independent genetic signals in the *ADIPOR2 *locus contribute to the risk of CVD and T2DM in a study population consisting of individuals with impaired glucose tolerance.

Of the eight SNPs analysed in DPS study population, rs10848554, rs11061937, rs1058322 and rs16928751 associated with the risk of CVD during the median follow-up of 10.2 years. When all four SNPs were included in the same model only rs11061937 and rs1058322 remained significant predictors of CVD, indicating independent effects of these two variants. This is supported also by the observation that these SNPs demonstrated a weak pairwise LD with each other. The rs11061937 was in low LD (r^2 ^ranging from 0.0090 to 0.071) with the other three SNPs, whereas rs1058322 was in moderate LD with rs10848554 (r^2 ^= 0.291) and rs16928751 (r^2 ^= 0.275), and rs10848554 and rs16928751 were in high LD with each other (r^2 ^= 0.918).

The rs16928751 variant has been previously found to be associated with plasma adiponectin and fasting triglycerides levels in a small study population of individuals with the metabolic syndrome [22], and T2DM in a case-control study [18]. Recently the association of an intronic variant, rs767870, with coronary artery disease, intima media thickness and endothelial dysfunction was reported in a cross-sectional study population [26]. This variant also associated with fasting plasma triglyceride concentrations, along with 13 other *ADIPOR2 *SNPs, in Mexican American subjects [23], and in a population based sample of 3050 Finnish subjects [24]. Moreover, rs767870 has been associated with measures of liver fat content and its surrogate markers [24], and T2DM in a case-control study of 1498 Caucasian subjects [20]. Unfortunately, rs767870 was not genotyped in our study population. However, in the HapMap CEU population, rs767870 is in high or moderate LD with three SNPs that associated with CVD risk in DPS: rs10848554 (r^2 ^= 0.774 and D' = 0.916), rs1058322 (r^2 ^= 0.337 and D' = 0.895), and rs16928751 (r^2 ^= 0.838 and D' = 1.0) [44]. None of the four variants associating with CVD risk have any known or obvious functional role. The rs16928751 is located in exon 7, but does not alter amino acid sequence, whereas both rs11061937 and rs1058322 are located in introns. Rs10848554 is located in the 5' promoter region and could potentially have some regulatory role in transcription regulation.

In the present study, the individuals homozygous for the rare minor alleles of two intronic SNPs, rs11061946 (T) and rs11061973 (A), had increased risk of conversion from IGT to T2DM. These SNPs are in high LD (r^2 ^= 0.674) and the individuals with the rs11061946 TT genotype were also the only ones who had the rs11061973 AA genotype. These results are in line with some of the earlier studies indicating association between other *ADIPOR2 *SNPs and the incidence of T2DM [18,20,21] even though a number of studies have failed to replicate association between *ADIPOR2 *SNPs and T2DM [27-29]. Both rs11061946 and rs11061973 are intronic SNPs, have no known functional significance, and may therefore be merely markers in LD with a true causal variant. Moreover, our results should be interpreted with caution, since the number of individuals homozygous for the minor alleles was low (n = 5), and genotype differences in T2DM risk were not observed when the dominant inheritance model was applied. The SNP rs1044471, located in the 3' untranslated region, did not associate with T2DM risk in the DPS population. Previously this SNP has been associated with T2DM in Amish [21], but not in Korean [28] population.

The regulation of the expression of *ADIPOR1 *and *ADIPOR2 *is currently poorly understood as findings in different tissues, species and physiological conditions are controversial. Several human studies have observed decreased AT expression of either or both receptors in obesity [17,45]. In muscle, the expression levels of *ADIPOR1 *and *ADIPOR2 *correlated positively with obesity, glucose and insulin levels and insulin resistance [46]. On the contrary, the expression levels were decreased in nondiabetic individuals with a family history of T2DM when compared with those without family history [15]. In liver, the mRNA levels of *ADIPOR2 *were increased [47] or decreased [48] in NASH, and the mRNA levels of both receptors were increased in insulin resistance [49]. Finally the expression of *ADIPOR1 *and *ADIPOR2 *at protein level was decreased in monocytes of individuals with coronary artery disease, but no difference was observed at the mRNA level [50].

These partly controversial results suggest that *ADIPOR1 *and *ADIPOR2 *expression may be regulated in a tissue-specific manner, and may be partly explained by genetic factors. Wang et al. found allele-specific differences in expression levels in individuals heterozygous for the *ADIPOR1 *SNP rs1139646 [51], whereas Halvatsiotis et al. observed that the *ADIPOR2 *variant rs767870 associated both with CVD and altered ADIPOR2 protein expression levels in peripheral monocytes [26]. Soccio et al. reported that *ADIPOR1 *SNPs predisposing to CAD were also associated with low *ADIPOR1 *expression levels in PBMCs and AT biopsies [52]. Interestingly, we found different expression levels according to rs1058322 genotype in middle aged Finnish individuals with impaired glucose metabolism and features of the metabolic syndrome who were participating in another study. Individuals carrying the T allele, who had increased risk of CVD in the DPS population, demonstrated decreased mRNA expression levels in the PBMC samples of the Genobin study participants [53].

The mechanisms by which *ADIPOR2 *variants might influence the risk of CVD and T2DM are currently hypothetical. The recent advances in human genetics have indicated that the gene variants underlying complex diseases usually have subtle effects on phenotype, often through temporal and spatial alterations in gene expression [54]. In addition, complex diseases and quantitative traits are largely affected by environmental factors that may interact with genetic factors. Moreover, genetic variants within the same gene locus or within different loci may have additive or epistatic effects on each other. These phenomena which are still poorly understood may explain to some extent the inconsistent findings in different study populations. Nevertheless, PBMCs are important players in inflammation, which is closely connected to both CVD and T2DM. Monocytes and macrophages are the target cells of the anti-atherogenic and anti-inflammatory effects of adiponectin [2,55] and SNPs affecting tissue-specific expression of *ADIPOR2 *may disturb these interactions directly.

The anti-atherogentic effects of adiponectin are well documented in vitro and in animal models [6,7,11,55] and circulating adiponectin levels are decreased in CAD patients [10]. Whether low adiponectin levels are associated with future risk of cardiovascular events is, however, currently unclear [56]. In a subgroup of DPS participants, we observed a trend for lower CVD risk in those with higher than median serum adiponectin levels compared with those whose adiponectin levels were lower than median. However, since the number of individuals with baseline measurements of circulating adiponectin data was low, this result should be interpreted with caution.

The limitation of this study is the fairly small size of the DPS population for genetic association studies. This may weaken the statistical power to find true associations or increase the chance of false positive findings. The associations between rs10848554, rs11061937, rs1058322, rs16928751 and the risk of CVD were significant when considering the combinations of p- and q-values (p < 0.05 and q < 0.150). However, although the p-values were significant the genotype associations with T2DM risk and mRNA expression levels the q-values were high and the results should be interpreted with caution. Concerning the expression studies, we measured *ADIPOR2 *expression only at mRNA level and it is not known if this difference is reflected in expression at protein level.

The major strength of this study is the longitudinal study design with a lifestyle intervention, which may increase the power to find true associations compared with cross-sectional settings. In addition, our study populations consisted of homogeneous group of individuals with impaired glucose metabolism, and therefore increased risk of both T2DM and CVD. Moreover, the risk of false association is further decreased by the accurate selection and phenotyping of the DPS participants. In addition we used the tagSNP selection approach to capture maximal amount of common genetic variation in the *ADIPOR2 *locus with minimal set of informative SNPs.

## Conclusions

In conclusion, the results of the present study are in agreement with previous findings suggesting a role for *ADIPOR2 *gene in susceptibility to CVD and T2DM, possibly through independent genetic effects. Different variants in the *ADIPOR2 *locus may act independently or in concert to induce subtle and possibly tissue specific alterations in gene expression. The observation of allele-specific differences according to rs1058322 variant in mRNA expression levels further support this view.

## Competing interests

The authors declare that they have no competing interests.

## Authors' contributions

NS participated in designing the genetic studies, performed the genotyping and statistical analyses, and drafted the manuscript. LP participated in designing the genetic studies and writing the manuscript. MK and US contributed to the design of the Genobin study and writing the manuscript. JL, JGE, PIP and SKK contributed to the DPS study design and coordination and revised the manuscript. JT and MU are the principle investigators of the DPS study and participated in writing the manuscript. All authors read and approved the final manuscript.
